# Promoting Positive Parenting Skills: A Systematic Review of Interventions

**DOI:** 10.1177/13591045261462246

**Published:** 2026-06-16

**Authors:** Inês Catarina Batista, Henrique Pereira

**Affiliations:** 1 Department of Psychology and Education (DPE), Faculty of Social Sciences and Humanities (FCSH), 56056 University of Beira Interior (UBI), Covilhã, Portugal; 2 Foundation for Science and Technology (FCT), Lisbon, Portugal; 3 Center for Research in Sport, Health and Human Development (CIDESD), Vila Real, Portugal

**Keywords:** parenting training, parenting skills, parent-child relationship, well-being, systematic literature review

## Abstract

Despite the increase in research in parenting skills, intervention programs, methods, and their evaluation remain segmented across different disciplines. This systematic literature review assessed and synthesized the existing literature on intervention programs aimed at parents or caregivers of children, and how they can promote positive parenting skills. Based on studies published between 2020 and 2025 in various databases (Web of Science, EBSCO, and Scopus), 13 peer-reviewed articles that fully met the inclusion criteria were analyzed. The results demonstrated that parental interventions, even with different formats and applied in diverse cultural contexts, are effective in improving parenting practices and, outcomes for children. Interventions based on psychoeducation, modeling, reinforcement, and personalized feedback proved to be the most effective. Future research may adopt a more inclusive approach regarding languages and publication sources.

## Plain Language Summary

This study analyzed what scientific research shows us about programs that help parents and caregivers develop positive parenting skills. These skills include communicating effectively, establishing consistent routines, and using disciplinary strategies that teach rather than punish. Many families face challenges in daily life, and these interventions aim to offer practical tools to improve the well-being of parents and children. To better understand the effectiveness of these interventions, we conducted a systematic literature review, in accordance with internationally recognized guidelines. We searched for articles published in various scientific databases and selected only studies that clearly evaluated their programs, participants, and outcomes. The included studies show that interventions in positive parenting skills are effective for families, even when presented in different formats or applied in different cultural contexts. Consistently, many parents or caregivers reported improvements in their communication with their children, greater confidence in their parenting abilities, and reduction in punitive practices. Several investigations also showed positive effects on children's behavior and emotional well-being. Despite the promising results, the review identified some limitations. In many of the programs, there was little representation of families considered to be at higher risk, and some studies faced difficulties with participation and full completion of activities. Some studies included few participants or short-term follow-up, which may also make it difficult to generalize the conclusions. Overall, the available evidence indicates that supporting parents through interventions focused on positive skills can be an effective way to promote children's development and strengthen family relationships. Investing in these programs can therefore benefit families in different contexts, from everyday family life to situations of greater vulnerability.

## Introduction

Parenting is a multidimensional process that plays a major role in shaping children’s developmental trajectories (Baumrind, 1971). Parental attitudes can promote children’s resilience, directly influencing how they face challenges and adjust to stressful situations (Caron et al., 2006; Gillespie et al., 2022; McKinney & Renk, 2008). These attitudes are often conceptualized as parenting styles (Darling & Steinberg, 1993), which can have effects on moral, interpersonal, and academic development (Bornstein & Marc Bornstein, 2007; Caron et al., 2006; Hill et al., 2004; Jiang et al., 2024; Pedro et al., 2015; Peh et al., 2020), as well as on children’s mental health (Caron et al., 2006; Hoeve et al., 2009). During early and middle childhood (approximately between 3 and 10 years of age), children undergo rapid development in terms of self-regulation, executive functions, language skills, and socio-emotional skills (Badarneh et al., 2024; Li-Grining et al., 2019; Prime et al., 2023). These skills, in turn, are sensitive to the quality of parent-child interactions, making this developmental phase particularly receptive to preventive parental interventions (LeCroy & Sullins, 2024; Sanders et al., 2014).

International organizations such as UNICEF point out that consolidating parenting skills is one of the most effective strategies for preventing behavioral disorders, promoting mental health, and reducing social disparities throughout life (Altafim et al., nd; UNICEF nd). In response, several programs promoting parenting skills have emerged, aiming to reduce coercive and neglectful practices (Lundahl et al., 2006), guiding parents towards a parenting style that promotes the psychosocial well-being of children and adolescents (Martinez-Escudero et al., 2020; Spera, 2005; Williams et al., 2024). These programs are structured with the goal of improving the quality of parenting and promoting positive interactions between parents and children, changing parental attitudes, knowledge, and practices (Fang et al., 2024; Masiran et al., 2020). This type of intervention offers caregivers alternatives to less supportive parenting practices and is among the evidence-based interventions with the greatest potential to reduce violence against children (Fang et al., 2024). All these programs are based on the coercive family process and social learning theory (Marquet-Doléac et al., 2024).

Parenting promotion programs therefore improve child development and behavior, parental mental health, parenting practices and, parent-child interaction (Fang et al., 2024; Li et al., 2024; Reichow et al., 2024). Additionally, they reveal effects on parental stress and feelings of parental efficacy (Marquet-Doléac et al., 2024; Reichow et al., 2024). Parenting interventions in preschool and elementary-school children can promote the child’s cognitive, motor, and socio-emotional development, parent-child bonding and interaction, parental knowledge, and positive parenting practices (Fang et al., 2024).

However, for intervention programs to be effective, it is essential to consider cultural and socioeconomic variables (Bornstein & Marc Bornstein, 2007; Hoff et al., 2002; Reichow et al., 2024). This way, a parenting program must be culturally adapted to be acceptable and relevant to different ethnocultural groups. Issues such as linguistic differences, cultural variations and program accessibility may limit its use (Masiran et al., 2020).

Numerous evidence-based parenting programs were analyzed and included in this systematic literature review (SLR). Despite emerging research in this framework, the literature remains methodologically divided. Studies vary in terms of design [randomized controlled trials (RCTs) vs. quasi-experimental studies], participant characteristics, measures of parenting assessment and child behavior, as well as the type of statistical analyses used (e.g., ANOVA, multilevel modeling, generalized linear models). This lack of methodological standardization complicates efforts to identify common patterns between interventions and to provide coherent recommendations for policies and practices. Furthermore, most systematic reviews were developed before 2020, focusing on specific populations with neurodevelopmental disorders or other clinical conditions. To fill these gaps and provide an up-to-date overview in the field of parenting, this SLR aims to:

- Analyze and catalog programs promoting positive parenting skills, carried out between 2020 and 2025, for parents of children aged 3 to 10 years, developed either face-to-face, online, hybrid, or with home visit formats;
- Evaluate the effectiveness of these programs on parenting skills and behaviors, parental well-being, child development, and behavior;
- Assess the methodological quality and potential sources of bias in the studies, applying standardized evaluation tools;
- Present mechanisms of change, therapeutic practices, and contextual factors related to the outcomes.

In this review, we present evidence from thirteen studies conducted in various countries, contributing to an understanding of how parental interventions can support families during early and middle childhood. Our aim is to help guide professionals who create, develop, and implement intervention programs; policymakers who may seek culturally adaptable solutions; and researchers dedicated to promoting theoretical and methodological coherence in the field of parenting science.

## Review Method

This SLR was conducted and reported in accordance with the guidelines of the PRISMA 2020 statement (Page et al., 2022). It was also pre-registered in PROSPERO (CRD420251046406).

### Criteria for Inclusion and Exclusion of Studies

The inclusion criteria were based on the PICO technique – Participants, Interventions, Comparisons, and Outcomes:

(a) Participants: the target audience had to consist of parents or caregivers of children aged 3 to 10 years. Studies conducted with pregnant women, involving only children and adolescents (without their parents), or studies with other target audiences were excluded.
(b) Interventions: All intervention programs focused on training positive parenting skills were included. However, studies that only reported the intervention protocol, without presenting the results, were excluded. Studies that focused on physical health or specific mental disorders were also excluded.
(c) Comparisons: Results should be compared to a control group. Studies that did not include a control group were excluded.
(d) Results: The results ought to include measures and analyses that demonstrate improvements in parenting skills, a decrease in problematic behaviors in children, and an improvement in the well-being of both parents and children. Interventions whose measures and analyses were not clearly reported or that did not present at least one measure or instrument for data analysis were not included.

### Data Collection Strategies

Data collection was carried out using the Web of Science, EBSCO, and Scopus databases, including studies published from 2020 to March 2025, to ensure a rigorous analysis and focus on the most recent literature – reflecting contemporary changes in parenting practices and programs. These databases were chosen because they allow research in different areas of knowledge. The initial search strategy, supported by the Medical Subject Headings (MeSH), included terms associated with parenting styles, parenting training, intervention programs and parenting skills, in both Portuguese and English. English was included in the research because it is a predominant language in publications in parental interventions and child mental health in general. Portuguese, in turn, was included due to the linguistic expertise of the research team and in an attempt to identify evidence in Portuguese-speaking contexts that might be underrepresented in international reviews. As recommended by the PRISMA guidelines, the complete search string was (e.g. Web of Science):

TS ((“estilos parentais” OR “parenting styles” OR “parental behavior” OR “child rearing” OR “parental attitudes”)

AND (“treino parental” OR “parenting training” OR “parenting education” OR “parental guidance” OR “parenting programs”)

AND (“programa de intervenção” OR “intervention program” OR “behavioral intervention” OR “educational programs”)

AND (“competências parentais” OR “parenting skills” OR “parental competence” OR “parental role” OR “parenting techniques” OR “parental effectiveness”))

AND LA = (English OR Portuguese)

AND PY = (2020–2025)

During the review process, after checking the set of articles identified in the three databases, we began analyzing the references of these articles to find additional studies. This “*snowball sampling*” method could allow us to find studies that the database search may have overlooked, with the goal of reducing selection bias and identifying a larger number of relevant studies on the theme. After checking the reference list of the included articles, no additional articles that met the inclusion criteria were identified. Articles that did not meet the inclusion criteria, as well as review articles, unpublished studies or grey literature, were removed from the results. To ensure the effectiveness of the search strategy, a validation process was conducted, which resulted in the identification of thirteen relevant studies.

### Article Selection Process

Data analysis was performed using the Mendeley Desktop tool. Before starting, duplicate references were removed and archived, first using the automatic feature of Mendeley Desktop and then manually. Titles and abstracts were then reviewed, and potentially relevant studies were included for full-text review. A PRISMA flowchart was created to report the number of studies identified (Figure 1).

**Figure 1.**
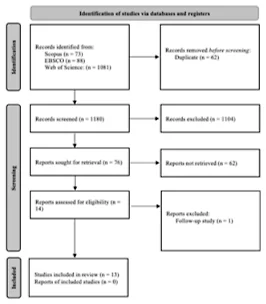
PRISMA flowchart

The initial search resulted in 1,242 studies. After automatic (*n* = 59) and manual (*n* = 3) removal of duplicates, the titles and abstracts of 1,180 studies were assessed for eligibility according to PICO criteria, and 1,104 were excluded. The main reasons for exclusion were: the target audience did not meet the criteria (e.g., children or adolescents, pregnant women, health professionals), interventions with a different objective (e.g., focus on physical health or specific mental disorders), studies with another type of design (e.g., SLR, quantitative, descriptive, observational, qualitative or correlational studies, that only seek to assess prevalences, protective and predictive factors, attitudes, experiences, or perceptions), studies with only a description of the intervention protocol, without presentation of the results, studies without a control group, studies that do not assess the impact on parenting skills or child behavior, and unpublished studies. The remaining 76 articles were included for full-text analysis, consequently excluding 62 studies. The reasons for exclusion at this stage were: the target audience did not meet the criteria (e.g., children under 3 years old or over 10), interventions with a different objective (e.g., focus on parental or sibling conflict), intervention with a very specific target audience (e.g., military families, families monitored by The National Commission for the Promotion of the Rights and the Protection of Children and Young People, families in military conflict contexts), the year of the study did not meet the criteria, and studies without full access. Finally, 14 studies appeared to meet the inclusion criteria, although 1 was excluded because it was a follow-up study associated with another article already included.

After selecting the studies, using an Excel spreadsheet, information such as title, authors, year of publication, country where the study was conducted, study design, characteristics of the intervention or program, characteristics of the participants, data collection and analysis tools used, main results, and limitations, were extracted. Table 1 presents a summary of the 13 included studies.

**Table 1.** Summary Table of Included Studies

ID	Study ID	Study design	Study participants	Type of intervention	Effect of intervention
S1	Winston LeCroy et al., 2024 USA	Randomized controlled trial	*N* = 664 families. Children aged 0–4 years. Majority female caregivers (97.6%).	Intervention based on home visits	Positive results in early childhood development, particularly in speech, hearing, and comprehension skills. Positive impact on parents who read more frequently to their children. Significant impact on parenting skills, such as effectiveness and hope.
S2	Breitenstein et al., 2021 USA	Randomized controlled trial	*N* = 287 parents or legal guardians. Children aged 2–5 years. Majority female caregivers (91.3%).	Online programs	The program may not be intensive enough for behavioral change in both parents and children. The program did not improve parent or child behavior. Parents in the intervention group reported less corporal punishment and improvements in parental affection, boundary setting, and proactive parenting.
S3	Brekke et al., 2023 Norway	Randomized controlled trial	*N* = 590 parents or guardians. Children aged 0–18 years. Majority female caregivers (∼75%).	In-person group programs	Significant positive effect in two outcome domains: parents and the parent-child relationship. Significant impact on children’s internalization problems and prosocial behavior. Increased closeness and reduced parental conflict.
S4	Carroll, 2022 USA	Quasi-experimental study	*N* = 91 parents and caregivers. Children aged 3–18 years. Majority female caregivers (81%).	In-person group programs	Participation in parenting workshops was associated with a decrease in authoritarian parenting style, a decrease in permissive parenting style, and a decrease in parental stress. It was also associated with an increase in the child’s academic competence and a decrease in externalizing behavior.
S5	Haar et al., 2023 Bangladesh and Indonesia	Quasi-experimental study	Bangladesh: *N* = 29 families. Children aged 11–15 years. Majority female caregivers (72%). Indonesia: *N* = 70 families. Children aged 8–14 years. Majority female caregivers (78%).	In-person group programs	Bangladesh: Significant improvements in positive reinforcement and parent-child relationship; significant increase in personal and global resilience in children in the intervention group. Indonesia: Significant reductions in coercion, increases in positive reinforcement, consistency, and parent-child relationship.
S6	Hutchings et al., 2023 UK	Randomized controlled trial	*N* = 56 caregivers. Children aged 3–8 years. Majority female caregivers (98% mothers).	Online programs	Significant improvements in reported parenting skills, with evidence of long-term maintenance. Increased parental praise, significant improvements in children’s behavior, increased parental effectiveness and satisfaction.
S7	Jespersen et al., 2021 USA	Inclusive randomized controlled trial	*N* = 213 primary caregivers. Children aged 0–5 years. Majority female caregivers (61% mothers).	In-person group programs	Pre- and post-test analyses showed significant increases in caregiver-reported responsive parenting, developmental knowledge, parenting efficacy, mindfulness, overall positive child behavior, prosocial child behavior, and reduced parental stress.
S8	Kirby et al., 2023 Australia	Randomized controlled trial	*N* = 102 caregivers. Children aged 3–9 years. Majority female caregivers (85.3%).	In-person group programs	Significantly reduced levels of self-criticism, significant reductions in children’s emotional problems and peer problems. There were no changes in parenting style. Significant improvement in self-compassionate actions.
S9	Linhares et al., 2022 Brazil	Randomized controlled trial	*N* = 92 mothers. Children aged 2–6 years. Only female caregivers (100%).	Hybrid interventions	Significant reduction in coercive practices, increased sense of parental competence. Significant reduction in behavioral problems in children. The reduction in childhood problems was mediated by the reduction in maternal coercive practices.
S10	Pontoppidan et al., 2024 Denmark	Quasi-experimental study	*N* = 510 families. Children aged 2–12 years. Majority female caregivers (∼92%).	In-person group programs	Parents in the intervention group felt more confident playing with their children, needed less help playing with their children, but reported that their children had more difficulty forming friendships. No significant effects on mental health, parental stress, self-efficacy, self-esteem, family routines, or child well-being. No significant effect on parental well-being.
S11	Sim et al., 2020 Australia	Randomized controlled trial	*N* = 355 caregivers. Children aged 8–11 years. Majority female caregivers (89%).	Online programs	Both control and intervention groups showed reductions in child anxiety and depressive symptoms, parental psychological control, and unhealthy family functioning; and improvements in parental acceptance and health-related quality of life for both children and parents. Contrary to expectations, reports of anxiety and depression by children and parents did not show significant differences between the groups. Improvements in the quality of life of children and parents, as well as in family functioning over time.
S12	Tuntipuchitanon et al., 2024 Thailand	Randomized controlled trial	*N* = 5,903 caregivers. Children aged 2–9 years. Majority female caregivers (82%).	Online programs	The program significantly improved parents’ sense of competence compared to the control group. Both groups showed a significant increase in authoritarian parenting styles and a decrease in permissive parenting styles. Both groups significantly reduced children’s emotional problems and overall behavioral problems.
S13	Wu et al., 2024 China	Quasi-experimental study	*N* = 139 parents. Children aged 3–6 years. Majority female caregivers (86.1%).	Hybrid interventions	Parents in the intervention group demonstrated lower levels of negative parenting behaviors after the intervention. Significant reduction in physical coercion and verbal hostility in the intervention group. There were no significant differences in behavioral problems between the groups.

### Study Characteristics

#### Design of Studies

The studies included in the present review were published between 2020 and 2025, conducted across multiple countries, including the USA (ID: S1; S2; S4; S7), Norway (ID: S3), Bangladesh and Indonesia (ID: S5), the United Kingdom (ID: S6), Australia (ID: S8; S11), Brazil (ID: S9), Denmark (ID: S10), Thailand (ID: S12) and China (ID: S13). Of the included studies, nine employed RCT designs, while four used quasi-experimental approaches (with experimental and control groups). The geographical and methodological diversity of the studies emphasizes the global relevance of parental interventions and the growing interest in measurable and evidence-based approaches to family support.

#### Characteristics of Participants

In the included studies, a total of 3,098 parents or caregivers were evaluated, with approximately half receiving some type of intervention to improve their parenting skills. We can estimate that a total of 530 participated in control groups, considering that some studies do not specify the number of participants allocated to the control group. The sample sizes of the studies ranged from small community-based cohorts (e.g., 29 families in Bangladesh) to large multicenter trials (e.g., 590 families in Norway). The studies primarily involved mothers, with fathers, grandparents, and other caregivers underrepresented across studies. The gender imbalance found in the included studies reflects the typical recruitment patterns in parenting research; however, it can limit the generalizability of the results. The participants had an average age between 30 and 40 years. Children’s ages ranged from 0 to 12 years, with most interventions targeting children at the early age range (3 to 6 years). The focus of studies on early and middle childhood reflects the ideal period for preventive intervention, considering the rapid developmental changes in children and their sensitivity to parenting practices. Most studies involve low- and middle-income families, although socioeconomic characteristics were inconsistently correlated.

#### Characteristics of Interventions

The studies were primarily categorized according to the delivery format, with a secondary analysis by type of prevention and focus of the intervention, which allows us to understand how the program’s structure can influence its effectiveness. Four main categories emerged – an initial set of six interventions (ID: S3; S4; S5; S7; S8; S10) was categorized as **in-person group programs**, with 4 to 12 sessions and trained facilitators; focusing on promoting positive parenting skills, improving parent-child relationships, and reducing stress and emotional difficulties for parents. A second group, consisting of four studies, was classified as **online programs**, subdivided into (a) self-guided online programs (ID: S2; S6; S11) in which parents or caregivers completed modules online or via a mobile app, without synchronous contact with facilitators; focusing on changes in parenting practices, parental stress, and child behavior problems; (b) synchronous online programs (ID: S12), with group sessions via video call; like face-to-face programs, but with some logistical flexibility. A third group, comprising two studies (ID: S9; S13), was considered as **hybrid interventions**, combining digital content with individualized contact. The last group, consisting of only one study (ID: S1), was considered an **intervention based on home visits**, focusing on promoting child development and providing guidance to parents.

#### Instruments for Data Collection and Analysis

The included studies used a variety of validated instruments to assess outcomes related to parental well-being, parental behavior, and child functioning. Parenting styles, skills, and behaviors, for example, were assessed primarily using the Parenting Stress Scale (*n* = 4), Sense of Competence Scale (*n* = 3), Parenting Styles and Dimensions Questionnaire (*n* = 2), and Young Children of Parents (*n* = 2). In turn, child development and behaviors were assessed using the Strengths and Difficulties Questionnaire (*n* = 7), the KIDSCREEN (*n* = 2), and The Eyberg Child Behavior Inventory (*n* = 2). Other instruments not mentioned were used only once. The heterogeneity of the instruments applied reflects different conceptualizations of parenting and child well-being; however, this complicates the direct comparison of results.

Across studies, the collected data was analyzed using robust statistical methods, with relevance to mixed (multilevel) models (ID: S2; S3; S4; S7), ANOVA/ANCOVA (ID: S1; S6; S7; S11), generalized linear models/GEE (ID: S9; S12; S13), simple correlation and regression (ID: S1; S4; S7; S11), effect size calculation (Cohen’s d) (ID: S1; S2; S3; S6; S9), and imputation methods for missing data (ID: S1; S2; S3; S7). Some trials employed intention-to-treat (ITT) or treatment-on-the-treated (TOT) approaches to address attrition and adherence (ID: S1; S3; S9).

### Quality of Studies

The Joanna Briggs Institute Critical Appraisal Tools (Joanna Briggs Institute (JBI), 2020a, 2020b) were used to assess the risk of bias in the studies included in this SLR. The JBI Checklist for RCTs was used for the nine randomized controlled trials included. For the four non-randomized studies, the JBI Checklist for Quasi-Experimental Studies was used. All included studies met more than half of the established requirements. This way, a balance was found between the inclusion of studies and the guarantee of quality and methodological rigor in our analysis.

In general, RCTs revealed robust allocation procedures and reliable outcome measures, although blinding was often not practicable considering the nature of behavioral interventions. In turn, quasi-experimental studies presented acceptable control conditions but were more vulnerable to selection bias. Common limitations among the studies included incomplete demographic descriptions, reliance on self-reported data, and short follow-up periods. Table 2 shows the requirements met for each of the studies and their respective scores.

**Table 2.** JBI Checklist for Randomized Controlled Trials (13 Items) and Checklist for Quasi-Experimental Studies (9 Items)

N	1	2	3	4	5	6	7	8	9	10	11	12	13
S1	X	#	X	X	o	#	X	X	X	X	X	X	X
S2	X	X	X	#	NA	X	X	X	X	X	X	X	X
S3	X	X	o	X	o	#	X	X	X	X	X	X	X
S6	X	X	X	#	NA	X	X	o	#	X	X	X	X
S7	X	#	X	X	o	#	X	o	#	X	X	X	X
S8	X	X	X	#	o	#	X	X	X	X	X	X	X
S9	X	#	X	#	o	X	X	o	X	X	X	X	X
S11	X	#	X	o	o	X	X	o	X	X	X	X	X
S12	X	X	X	X	o	X	X	X	X	X	X	X	X
S4	X	X	#	X	X	o	X	X	X	​	​	​	​
S5	X	o	X	X	X	X	X	X	X	​	​	​	​
S10	X	o	X	X	X	o	X	X	X	​	​	​	​
S13	X	X	X	X	X	o	X	X	X	​	​	​	​

Met requirement (X), didn’t meet requirement (o), unclear (#), not applicable (NA).

## Results

### General Description of the Included Studies

#### In-Person Group Programs

Six interventions were found that were delivered through structured, in-person group sessions, facilitated by trained professionals or community agents. Programs such as the International Child Development Program (ICDP), Positive Discipline in Everyday Parenting, Family UNited, and Active Parenting focus on consolidating emotional communication, promoting non-punitive discipline, and improving parent-child relationships (Brekke et al., 2023; Carroll, 2022; Haar et al., 2023; Jespersen et al., 2021; Kirby et al., 2023; Pontoppidan et al., 2024). These interventions are developed in 4 to 12 weekly sessions, combining instructional content with opportunities for modeling, role-playing, and/or peer interaction.

The International Child Development Programme (ICDP) (Brekke et al., 2023) is a universal parenting program focused on positive communication and promoting child development, implemented in family-friendly settings within parent groups. The program consists of several group sessions held over a period of several weeks. The intervention is delivered by facilitators with specific program training (not necessarily psychologists). The ICDP has demonstrated improvements in indicators of parenting practices (greater sensitivity, fewer negative practices) and some indicators of parental well-being, with more modest effects on child behavior.

In turn, Positive Discipline in Everyday Parenting (Carroll, 2022) is a positive discipline program implemented in a community setting with low-income families. It focuses on non-punitive strategies, problem-solving, promoting mutual respect, and socio-emotional skills. The program consists of multiple weekly sessions (≈7–8 weeks), in a workshop format. The program has reduced the use of physical/verbal punishment and increased the use of positive and problem-solving strategies, with moderate improvements in child behavior.

For its part, Family UNited (Haar et al., 2023) consists of a universal family prevention program based on parenting skills, family communication, and risk prevention. Approximately 8 sessions are implemented, combining sessions for caregivers only, children only, and family sessions. These sessions are conducted by trained community facilitators, often NGO technicians or health/community professionals trained in the program. The program has demonstrated improvements in positive parenting practices and family communication, with a reduction in rigid discipline practices and some gains in child behavior.

The Active Parenting: First Five Years (Jespersen et al., 2021) program is an educational parenting program for parents of children aged 0 to 5 years, focusing on developmental understanding, positive discipline techniques, and the promotion of socio-emotional skills. The program is typically delivered in four 2-h group sessions, facilitated by municipal service professionals with specific training in the program. Parents reported improvements in parental self-efficacy and the use of positive strategies, with small to moderate effects on coercive practices and parental stress.

The brief Compassion-Focused Parenting Workshop (Kirby et al., 2023) is a single group session based on Compassion-Focused Therapy (CFT), focusing on parental self-compassion, regulation of shame/self-criticism, and emotional responsiveness in the relationship with the child. The session lasts approximately 2 hours and is delivered by clinical psychologists or therapists trained in CFT. This single session produced relevant reductions in parental self-criticism and shame and increases in self-compassion and parental warmth; however, the effects on child behavior were more modest.

Finally, the Family Club Denmark (Pontoppidan et al., 2024) consists of a voluntary, community-based intervention with bi-weekly family clubs, focusing on togetherness (shared meals), play, and building social networks, applying principles of positive psychology and social learning. The clubs meet every 2 weeks for 6 months (≈12 sessions), led by a team of volunteers, based on four core principles – community at the table, play/learn, support/advice, connection with civil society. In vulnerable families, the program increased parents’ confidence to play with their children and reduced the need for help with play; however, it did not show consistent effects on mental health, parental stress, family routines, or child well-being.

After analyzing these studies, the results indicate that in-person group programs produce moderate to significant improvements in parenting skills, particularly in terms of increased positive reinforcement, consistency, and emotional responsiveness. Several studies have also documented improvements in parent-child closeness and modest reductions in children’s behavioral difficulties. Compassion-based programs, such as the brief Compassion-Focused Parenting Workshop, produced significant gains in parental self-compassion and emotional regulation, although the effects on children’s outcomes were smaller.

#### Online Programs

Four studies evaluated online interventions. Three of these studies were self-guided programs (ezParent, COPING – COnfident Parent INternet Guide, PaRK/PaRC) (Breitenstein et al., 2021; Hutchings et al., 2023; Sim et al., 2020) and one study evaluated a synchronous program, conducted by a facilitator (Triple P – Positive Parenting Program) (Tuntipuchitanon et al., 2024).

The ezParent program (Breitenstein et al., 2021) consists of a mobile application for positive parenting (ezParent), based on the Chicago Parent Program, with several videos and exercises focused on positive practices, supervision, and consistent discipline. Six self-guided modules are available to caregivers, which they should complete at their own pace over several weeks. The ezParent program has shown improvements in positive parenting practices and a reduction in negative practices, also achieving some effects on children’s behavioral problems.

In turn, COPING – COnfident Parent INternet Guide (Hutchings et al., 2023) is a universal 10-week self-guided program for parents of children aged 3 to 8 years, based on principles of social learning theory. It includes content and review modules, with modeling videos, quizzes, and practical tasks. This study was a pilot feasibility trial and, therefore, there were recruitment difficulties and a high dropout rate; however, among the parents who participated, improvements in parenting practices were observed (more praise, fewer vague commands) as well as some reduction in problematic children’s behavior.

Finally, the PaRK/PaRC program (Sim et al., 2020) is a self-guided online intervention focused on responsive parenting (routines, emotional and behavioral responsiveness, management of common challenges), with some interactive modules. The intervention produced improvements in parental responsiveness and disciplinary consistency, with significant reductions in behavioral problems reported by parents, compared to the control group.

Self-guided interventions produced small to moderate improvements in parenting behaviors, particularly regarding reductions in punitive disciplinary practices and improvements in parental affection and responsiveness. However, the results for children were more variable, and effectiveness depended heavily on parental involvement and module completion rates, with attrition and limited interaction with facilitators being identified as barriers to their effectiveness.

Only the Triple P – Positive Parenting Program via Zoom (Tuntipuchitanon et al., 2024) is a synchronous online version of the Group Triple P, with live video call sessions, maintaining the content and structure of the original manual. Parents participated in a series of group sessions, facilitated by therapists certified in the Triple P program, who conducted the live group sessions via the online platform. The synchronous version of the Triple P – Positive Parenting Program demonstrated significant results, with substantial improvements in parenting skills and a reduction in children’s behavioral problems as well as parental stress. This might suggest that real-time interaction can preserve the relational elements of the traditional group delivery.

#### Hybrid Interventions

Hybrid programs, such as The Strengthening Bonds (based on video feedback) and the school program that combines video and telephone, integrate digital content with personalized and ongoing support from facilitators (Linhares et al., 2022; Wu et al., 2024).

The Strengthening Bonds (Linhares et al., 2022) is a universal preventative program focused on personalized remote video feedback to promote positive parenting practices and reduce problematic childhood behaviors. It consists of one in-person group session with up to 10 mothers, addressing concepts of parenting, development, and positive interaction. This is followed by six weeks of personalized video feedback sent via WhatsApp, with edited videos of mother-child interactions in play situations. The psychologist conducts the in-person session and subsequently prepares and sends the commented videos. Intention-to-treat analysis showed no significant overall effects; however, treatment-on-the-treated analysis showed significant reductions in coercive maternal practices and children’s behavioral problems, as well as an increase in parental competence, with the reduction in coercive practices mediating the decrease in children’s problems.

In turn, the school program with videos and phone calls (Wu et al., 2024) is a hybrid school-home program in which parents receive training videos and follow-up phone calls. This program focuses on positive parenting practices and support for learning and school behavior, combining video series and structured phone calls. The program is facilitated by professionals linked to schools and health services, who supervise access to materials and conduct the guidance calls. The study found improvements in parenting practices and school engagement, and reductions in some behavioral problems, especially for families who completed a greater number of contacts.

These methodologies employed both accessibility and personalized guidance, resulting in significant reductions in coercive parenting practices and improvements in child behavior, particularly among families that maintained consistent engagement. The use of personalized video feedback was particularly efficient in helping parents reflect on real-life interactions and assume more positive strategies.

#### Intervention Based on Home Visits

Parents as Teachers (PAT) (Winston LeCroy et al., 2024) is a home-visiting program based on the PAT model, focusing on child development, responsive parenting practices, and service engagement, comparing a standard model and an enhanced model. Regular visits are conducted throughout the child’s first years of life by home visitors – parent educators – trained in the PAT model. This program has revealed significant improvements in outcomes such as parental knowledge, parenting practices, child development, and school readiness. However, the intensity and resources required for home visits may limit their scalability compared to online or group models.

### General Description of the Assessed Outcomes

#### Parenting Skills, Behaviors and Well-Being

In the interventions considered, improvements in positive parenting practices were the most consistent results, regardless of the delivery format. Increases in the level of warmth, praise, stability, and parental proactivity were reported in most studies. Reductions in negative parenting behaviors, such as coercion, punitive discipline, verbal hostility, and psychological control, were also often noted.

#### Child Behavioral and Emotional Outcomes

Child outcomes were generally positive, but more variable compared to the outcomes for the parents. Various programs demonstrated moderate reductions in children’s externalizing and internalizing behaviors, particularly when coercive parental practices decreased significantly. However, some studies found no differences between groups in terms of child behavior, which may suggest that improvements in children may require a higher intensity of intervention or a longer duration.

#### Parent-Child Relationship Quality

In several programs, improvements were observed in the relational dynamics between parents and children, particularly in terms of conflict reduction, increased closeness, and greater mutual understanding. Programs that used emotional communication frameworks or family sessions (e.g. ICDP) showed significant effects in this area.

Positive changes were, also, observed in the well-being and stress of parents or caregivers, although the results varied according to the program format. Improvements in parental self-efficacy were more consistent, particularly in hybrid and in-person interventions. Compassion-based approaches contributed uniquely to reducing parental self-criticism and emotional dysregulation.

## Discussion

This SLR aims to summarize the most recent evidence regarding interventions designed to promote positive parenting skills in parents or caregivers of children aged 3 to 10 years. The thirteen studies included in this review demonstrate that parenting interventions, regardless of their delivery format, are largely effective in improving parenting practices and, to a lesser extent, children’s socio-emotional skills and behavior. The results show an increasing variation in program delivery formats, as well as the benefits of interventions based on well-established theoretical frameworks and the challenges posed by the methodological variability of each study. We now aggregate these findings to explore the mechanisms by which interventions achieve their effects, assess the heterogeneity between different formats and contexts, and identify future directions for research and public policy.

### The Effects of the Interventions

The in-person and hybrid interventions analyzed obtain the most significant results, while online programs, despite offering promising advantages, present more variable results. Individualized guidance, and the theoretical foundation of the programs seem to emerge as common factors in the most efficient interventions. Although some methodological variability exists, the results confirm the importance of parenting intervention programs.

In-person interventions (e.g., ICDP, Positive Discipline, Family UNited) generally show moderate to significant improvements in parenting skills, parent-child closeness, parental self-compassion, and emotional regulation, although the effects on children are smaller (Brekke et al., 2023; Carroll, 2022; Haar et al., 2023; Jespersen et al., 2021; Kirby et al., 2023; Pontoppidan et al., 2024). These results are consistent with research in the field indicating that structured group formats consistently improve parenting skills through modeling, facilitator feedback, and peer social support (Lyu et al., 2023; Mathijs et al., 2024). However, results on child behavior tend to vary more. Some interventions have effectively demonstrated improvements in children’s emotional and behavioral functioning (Carroll, 2022; Haar et al., 2023), while other studies have shown modest or inconsistent changes. Despite their effectiveness, in-person programs have some limitations, as they require multiple sessions, trained facilitators, and infrastructure. Accessibility to them can also be restricted, particularly for families facing socioeconomic difficulties or geographic isolation, leading to concerns regarding program equity.

Online interventions have yielded more varied results. Programs such as ezParent, COPING, and PaRK/PaRC have shown improvements in parenting skills and a decrease in negative behaviors, but the effects are mostly dependent on participant engagement (Breitenstein et al., 2021; Hutchings et al., 2023; Sim et al., 2020). This variability may raise several issues, such as dropout rates, especially in self-guided formats; the lack of contact with a facilitator, which may reduce opportunities for individualized guidance; and the need for participants to have some digital literacy and access to resources that some families do not benefit from. In turn, programs such as the Triple P – Positive Parenting Program via Zoom, in synchronous format, show more consistent improvements in terms of parenting skills and children’s behavior (Tuntipuchitanon et al., 2024), which may indicate that real-time interaction with facilitators can be central to preserving some of the relational benefits of group-based models. In general, online intervention programs can prove to be a relevant expansion in the provision of services to families.

Hybrid interventions, which integrate digital content with personalized feedback (e.g., The Strengthening Bonds, School program with videos and phone calls), have shown notable effects, especially when video feedback allows for learning through observation and reflection (Linhares et al., 2022; Wu et al., 2024). These programs benefit from the flexibility of digital technology while maintaining individualized support, which seems to consolidate parental trust, reduce coercive parenting practices, and improve children’s behavioral problems. Similarly, programs with home visits, such as PAT, demonstrate significant improvements in promoting child development and parenting skills (Winston LeCroy et al., 2024). Despite requiring considerable resources, home visits constitute an effective intervention strategy in early childhood (Hilário et al., 2022), offering personalized support adjusted to the context. Thus, hybrid and home-based approaches are effective in strengthening parent-child relationships, as they allow for individualized feedback.

### Mechanisms to Promote Parenting Skills

In the studies evaluated in this review, several mechanisms appear as contributors to positive results –modeling and reinforcement, parental self-efficacy, emotional self-regulation and self-compassion, communication, and structured and consistent routines. Models based on social learning (e.g., Triple P – Positive Parenting Program, COPING, ezParent) enhance parenting skills by demonstrating positive behaviors and providing structured practice opportunities (Breitenstein et al., 2021; Hutchings et al., 2023; Tuntipuchitanon et al., 2024). Additionally, interventions such as Active Parenting, The Strengthening Bonds, and PAT increase parents’ sense of competence, which is strongly associated with parental and child behaviors (Jespersen et al., 2021; Linhares et al., 2022; Winston LeCroy et al., 2024). In turn, the brief Compassion-Focused Parenting Workshop highlighted emotional self-regulation as central to helping parents cope with frustration, reducing self-criticism, and respond more positively to children’s behavior (Kirby et al., 2023). Programs such as ICDP and Family United have reinforced parents’ sensitivity and understanding of their children’s developmental needs (Brekke et al., 2023; Haar et al., 2023). Finally, programs such as COPING and PaRK/PaRC have fostered consistent discipline and the establishment of routines (Hutchings et al., 2023; Sim et al., 2020).

### Cultural and Contextual Adaptation of Interventions

One of the strengths of this SLR lies in the vast international scope of the included studies, encompassing multiple global regions. This multiplicity of contexts allows for a greater understanding of how culture can influence parenting practices and the effectiveness of interventions. For example, Bangladesh and Indonesia appear to report significant results in positive reinforcement and family resilience (Haar et al., 2023), which may reflect collectivist values and extended family structures (Pelham et al., 2022). In China, the school program with videos and phone calls led to decreases in severe disciplinary practices, aligning with shifts toward less authoritarian parenting in these contexts (Wu et al., 2024).

Despite the development of these studies, cultural adaptation remains a significant challenge. Programs established in Western countries may require translation, contextualization, and facilitator training to ensure cultural relevance. Furthermore, since most samples are predominantly female, they reflect existing gender expectations regarding childcare, possibly limiting generalizations to other caregivers (e.g., fathers, grandparents).

## Limitations and Future Directions

The studies analyzed present some limitations that may hinder the interpretation of the results, namely the fact that the samples are predominantly female, the underrepresentation of families experiencing multiple psychological stressors, the limited descriptions of the socioeconomic level of the families, as well as issues of participant engagement and fidelity, particularly in programs developed online. Furthermore, some studies have small sample sizes, which may limit the statistical power of the results. Moreover, most studies described results immediately after the intervention, with few long-term follow-ups, which can make the maintenance of the results unclear. Additionally, the studies included used different tools to assess parenting skills, parental well-being, and children’s behavior. This heterogeneity of measures and methods applied may limit direct comparison and the potential for meta-analysis, consequently restricting the synthesis of the results (Deeks et al., 2024), highlighting the need for standardized measures.

In addition to the limitations of the studies included, this SLR also presents some limitations that should be considered while interpreting the results. The restriction of the chosen languages for the search may have limited the inclusion of relevant studies. Similarly, the selection of the databases for our search can also be considered a limitation, since studies indexed in other databases may have been overlooked.

To overcome the aforementioned limitations, future research should consider implementing a more inclusive approach regarding language and publication sources. Expanding research beyond publications exclusively in English can contribute to a broader and more culturally representative understanding of parenting interventions in different regions. Future SLR could also benefit from international and multilingual collaborations, which could facilitate access to studies in other languages, reduce language bias, and support cultural adaptation of evidence-based parenting programs. Studies should include long-term or longitudinal follow-up studies, to ascertain the maintenance of results (Deeks et al., 2024; Fogel, 2018; Kazdin, 2017). Furthermore, it will be important to apply standardized outcome measures to facilitate comparison between interventions and different contexts. Similarly, the sample size and diversity of participants should be increased, including other types of caregivers such as fathers, grandparents, same-sex parents, and families with greater socioeconomic or cultural diversity, to ensure greater representativeness. Additionally, it may be interesting to conduct comparative studies, directly comparing the type of program delivery format (e.g., in-person vs. online vs. hybrid), to determine its effectiveness.

## Implications for Practice

Our SLR results suggest that intervention flexibility is crucial when working with families. Offering different delivery formats for intervention programs can help overcome some difficulties, meeting families’ needs and reducing barriers to access. Interventions based on psychoeducation, modeling, reinforcement, and personalized feedback proved to be the most effective, with hybrid models, which incorporate video feedback, being among the most favored. To achieve this, formats that integrate online components should include strategies that address families’ limited access to technology. We also found that facilitator training is fundamental to the success of the intervention, with programs delivered by trained professionals under continuous supervision tending to yield better results. Parenting skills development programs should intentionally involve fathers and other caregivers to maximize their impact.

These groundbreaking findings can provide an evidence base that strengthens the clinical practice of healthcare professionals, incorporating these approaches into family mental health programs and policies, facilitating the development of upcoming interventions and increasing positive outcomes in this population.

## Data Availability

To request the data extracted for this systematic literature review, please send an email to Inês Catarina Batista at inescmbatista@gmail.com. The data will be provided, upon request, for academic or research purposes, and only if the ethics and privacy policies of both the institution and the research project are respected.
